# Measuring visfatin levels in saliva: an alternative approach to gestational diabetes screening

**DOI:** 10.20945/2359-3997000000396

**Published:** 2021-11-11

**Authors:** Hacer Eroglu İÇLİ, Tevfik Berk BİLDACI

**Affiliations:** 1 Republic of Turkey Ministry of Health Sultan Abdulhamid Han Education and Research Hospital Division of Biochemistry Istanbul Turkey Republic of Turkey Ministry of Health, Sultan Abdulhamid Han Education and Research Hospital, Division of Biochemistry, Istanbul, Turkey; 2 Baskent University Obstetrics and Gynecology Department Istanbul Education and Research Hospital Istanbul Turkey Baskent University Obstetrics and Gynecology Department, Istanbul Education and Research Hospital, Istanbul, Turkey

**Keywords:** Adipokine, gestational diabetes, oral glucose tolerance testing, visfatin

## Abstract

**Objective:**

Oral glucose tolerance testing (OGTT) is the current recommended approach for the diagnosis of gestational diabetes mellitus (GDM). Visfatin is a type of novel adipokine of interest that mostly participates in glucose metabolism and inflammatory processes. We aim to identify a screening technique for GDM using salivary visfatin levels and to establish this technique’s value as a screening method compared to OGTT.

**Materials and methods:**

This is a cross-sectional case-control study. The cohort was formed from the saliva samples of pregnant patients in their 24^th^ through 28^th^ weeks of gestation. Patients were divided into two groups depending on their GDM status. OGTT and visfatin test results were compared and subjected to further analysis to establish a cutoff value for visfatin testing.

**Results:**

ELISA results indicated a significant difference between patients with GDM compared to patients without GDM; the values were 18.89 ± 9.59 and 12.44 ± 8.75, respectively (p: 0.007). A cutoff value of 10.5 ng/mL can be used to detect GDM with 78% sensitivity and 51% specificity.

**Conclusion:**

Salivary visfatin levels were significantly higher in patients with GDM. The existence of a differential in the concentration of visfatin in saliva can be utilized to develop a new screening method for GDM.

## INTRODUCTION

During pregnancy, patients are prone to many complications that can emerge during this period, such as gestational diabetes mellitus (GDM). GDM is a common complication defined as glucose intolerance initiated during pregnancy with no previous history of glucose intolerance. Following the adoption of International Association of Diabetes and Pregnancy Study Group’s recommendations, the prevalence of GDM in developed countries was found to be around 18%, an increase of about 40% from previous estimates between 1989 and 2004 (1,2). Oral glucose tolerance testing (OGTT) is an accepted method of testing and identifying glucose intolerance in pregnant patients that is recommended by various organizations such as the American College of Obstetrics and Gynecology (ACOG) and the American Diabetes Association (ADA) (3,4).

Adipokines are peptides and proteins with various functions. They generally perform various tasks related to vascular hemostasis and blood pressure regulation (5). Adipose tissue is an active endocrine organ that plays a substantial role in the specific hormone production of adipokines such as visfatin, which are believed to participate in energy storage. Dysregulation of pathways involving visfatin peptides has been observed to cause alterations in insulin sensitivity (6).

Visfatin can be encountered throughout the literature under various alternative names such as Pre-B-cell colony-enhancing factor 1 (PBEF1) and nicotinamide phosphoribosyl transferase (NAMPT). PBEF1 is a protein originating from visceral adipose tissue that exists in both humans and mice, and which is a contributing factor to the rate-limiting step in the nicotinamide adenine dinucleotide (NAD+) salvage pathway. This pathway plays a role in converting nicotinamide to nicotinamide mononucleotide to enable NAD+ biosynthesis (7,8). Expression of visfatin is also observed in placental tissue, but in significantly lower quantities compared to its expression in subcutaneous and visceral adipose tissue. Visfatin secretion is strongly influenced by blood glucose levels in humans. Obesity is another factor that is associated with increased visfatin levels through an insulin-like effect in which it binds to the insulin receptor-1 and where hypoglycemia is acquired through mechanisms involving the reduction of glycogenolysis and stimulation of glucose utilization (9,10). Circulating visfatin levels are increased in patients with type 2 diabetes, metabolic syndrome, and/or cardiovascular diseases (11).

Adipokines can also be detected in saliva. A limited number of studies show a positive correlation between salivary levels of visfatin and other adipokines such as ghrelin and resistin in patients with type 2 diabetes mellitus (12). It was also recently established that levels of adipokines, including visfatin, are positively correlated with inflammatory conditions involving periodontal tissue, such as chronic periodontitis (13,14). In this study, our goal is to identify whether a relationship exist between salivary visfatin levels and OGTT results showing GDM or a lack thereof.

## MATERIALS AND METHODS

**Figure 1:**
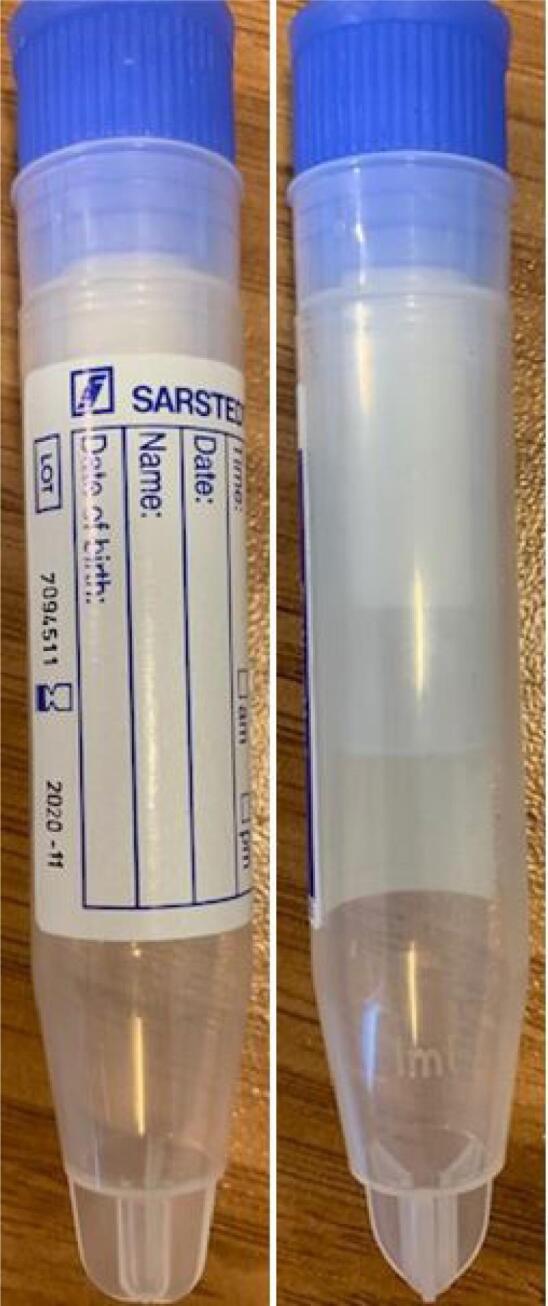
Images of saliva collection tubes.

This is cross-sectional, case-control study was performed at Baskent University Istanbul Education and Research Hospital. The cohort was formed from saliva samples of patients in between their 24^th^ through 28^th^ weeks of pregnancy. Samples were collected using specialized collection tubes (Salivette^®^, Sarstedt AG & Co., Germany) (Figure 1) prior to initiating the patients’ OGTT. Patients who were present for their routine prenatal visit for GDM screening in a low-risk clinical setting were asked to participate in this study during the allocated period. Collection time varied between 9 AM and 11 AM. Prior to saliva collection, patients were examined for signs of gingivitis by a dental practitioner with 5+ years of experience in field. Patients found to have gingivitis were excluded from the study. Saliva samples were collected during a 3-minute time frame using the collection tubes cited earlier. All saliva samples were centrifuged at 1000G for 20 minutes at temperatures between 2 °C and 8 °C. This saliva supernatant was divided into 1-mL aliquots in cryotubes. Specimens were immediately frozen to a temperature of -80 °C and kept under same conditions until adipocytokine analysis. Immediately before the analysis, samples were thawed to room temperature, or 22 °C.

The presence of GDM was assessed using 75-g oral glucose solution and diagnosed when any one of the following three results were identified: fasting ≥ 92 mg/dL (5.1 mmol/L), 1-hour glucose level ≥ 180 mg/dL (10.0 mmol/L), or 2-hour glucose level ≥ 153 mg/dL (8.5 mmol/L). Patients diagnosed with GDM were initially treated with nutritional recommendations by a dietitian, if appropriate. If nutritional therapy was found to be ineffective or inadequate for achieving the target glucose levels (i.e., fasting ≤ 95 mg/dL, 1 hour ≤ 140 mg/dL, and 2 hours ≤ 120 mg/dL), insulin therapy was initiated in consultation with an endocrinology specialist.

Visfatin levels were tested using an enzyme-linked immunosorbent assay (ELISA) kit (Bioassay Technology Laboratory/Cat no. E0025, Hu Shanghai Korain Biotech Co., Ltd., Shanghai 200090, China). Inter- and intra-assay coefficients of variation (CV) were calculated to be < 10% and < 10%, respectively. We preferred studying saliva samples rather than serum because of the novelty of salivary assessment and patients’ desire for an easily applicable, non-invasive testing choice on GDM diagnosis.

In total, 91 patients provided appropriate consent for their samples’ use in the study. This study was deemed in compliance and approved by the Baskent University Ethical Committee as KA19/395. SPSS version 23.0 was used for statistical analysis (IBM Corp. Released 2015. IBM SPSS Statistics for Windows, Version 23.0. Armonk, NY: IBM Corp.). An independent Student’s t-test, Pearson correlation analyses, a ROC curve, and an AUC analysis were used where appropriate.

## RESULTS

Patients diagnosed with GDM were assigned to the study group (SG), which included 18 patients, whereas patients with normal OGTT values were assigned to the control group (CG), which included 73 patients. We lost touch with 17 patients during pregnancy follow-ups, so their childbirth outcomes were not included in the analysis. A post hoc analysis was performed following saliva sample collection, and a power of 0.92 with an effect size of 0.8 was achieved with the two groups’ sample sizes. The incidence of GDM was 19.8%. The mean age of the SG was 34.84, and the mean age of the CG was 32.05 (p: 0.035). Mean of patient properties and birth related outcomes were outlined in Table 1.

**Table 1:** Mean comparisons of patients’ study-relevant properties and childbirth data

	Study Group (SG) n: 18	Control Group (CG) n: 73	Significance (p)*
Age	34.83 ± 4.95	32.05 ± 4.93	**.035**
Body mass ındex (BMI)	28.18 ± 2.68	27.06 ± 2.77	.125
Gestational weeks at study	25.47 ± 1.14	25.08 ± 1.38	.269
Birth week (CG missing 17 patients)	38.23 ± 1.47	38.62 ± 1.12	.233
Birth weight in grams (CG missing 17 patients)	3297 ± 521	3244 ± 365	.633

* Significant values are indicated in bold.

The ELISA results showed a significant difference between the two groups; SG: 18.89 ± 9.59 ng/mL and CG: 12.44 ± 8.75 ng/mL (p: 0.007). When age was tallied as an effecting cofactor, there was still a significant difference between two groups, as p: 0.009 in a controlled analysis. When OGTT results are analyzed individually for every hour and for fasting values, among the three values tested during the OGTT, only the 1-hour blood glucose level correlated significantly with visfatin levels. The results of the correlation analyses are listed in Table 2.

**Table 2:** Independent correlation of visfatin values with OGTT values

Correlations	Visfatin	Fasting Glucose	Glucose First Hour	Glucose Second Hour
Visfatin	1	.102	.214	.183
Fasting Glucose	.137	1	**.520**	**.391**
Glucose First Hour	**.211**	**.520**	1	**.762**
Glucose Second Hour	.180	**.391**	**.762**	1

* Significant values are indicated in bold (significance level < 0.05).

**Figure 2:**
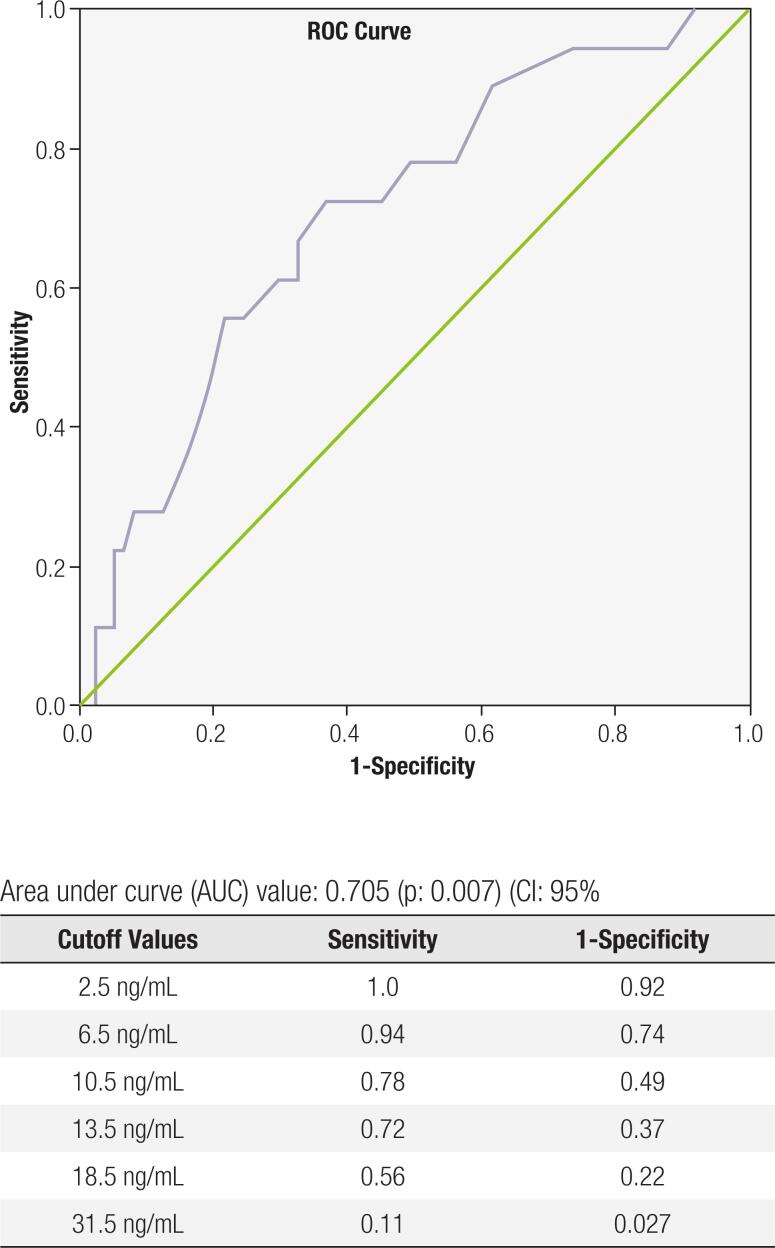
ROC Curve and AUC analyses for visfatin values used to identify GDM.

ROC (receiver operating characteristics) curve and AUC (area under curve) analyses were performed to identify a reasonable cutoff value for visfatin levels. AUC was observed to be 0.705, with significance level of p: 0.007 and 95% CI (0.574 and 0.836 – lower and upper bounds, respectively). Using the point closest to the upper left corner of the chart, where sensitivity and specificity are greatest at their own values, 13.5 ng/mL was set as a cutoff value, achieving a sensitivity of 72% and a specificity of 63%. Other sample values for the cited specifications are shared in Figure 2.

## DISCUSSION

The relatively high prevalence of GDM, which ranges from 18% to 30% and increases in likelihood with a patient’s age, makes this issue a priority, especially considering its associated negative economic impact and potential severe pregnancy complications (15). We observed an incidence of 19.8% among our patients, with a significant age difference between groups similar to that observed in a previous study of ours (16).

Even though treating GDM decreases the risk of associated complications, making the diagnosis as early as possible in pregnancy is of extreme importance. To overcome the heterogeneity in the diagnosis, universal OGTT based screening is employed to identify pregnant women with GDM. In general, adipokines, which play significant roles in glucose metabolism, can be utilized in the diagnosis of patients with GDM. Although their secretion might be altered by obesity, potentially making adipokine studies unreliable, numerous other studies have identified significant variations in adipokine levels in patients with GDM (17-19). Because studies involving adipokines were mostly performed using serum samples, our study employs a novel approach by using saliva samples to diagnose GDM.

The role of visfatin in the human body is not clearly established. However, these adipokines are involved in the regulation of energy homeostasis, which is the pathogenesis of diabetes mellitus. A strong meta-analysis by Chang and cols. showed that plasma visfatin concentrations were elevated in patients diagnosed with type 2 diabetes mellitus, metabolic syndrome, and cardiovascular diseases (20). In addition, Mopidevi and cols. observed that visfatin levels decreased following periodontitis treatment (13). Because the inclusion of patients with gingivitis would create a bias, we chose to exclude patients diagnosed with gingivitis. As the samples were collected from saliva, the matter of periodontitis was of utmost importance for us.

In a case-control study by Karatas and cols. that examined gestational weeks similar to those in our study, visfatin values detected in serum were not significantly different between patients with and without GDM (21). Because visfatin is secreted by epithelial cells of the amniotic sac during pregnancy, another group of researchers tested visfatin serum levels in patients in their 12th through 15th weeks of pregnancy and was unable to identify a significant difference between patients who later developed GDM and those who did not (22,23). However, Rezvan and cols. found significantly lower serum levels of visfatin in patients using OGTT (24). Therefore, we can assume that data about serum levels for visfatin and their use in establishing glucose intolerance remain controversial in the literature. This is one of the reasons we conducted this study on a novel technique using saliva samples.

A recent study of patients in early pregnancy performed by Bawah and cols. observed that visfatin, along with other adipokines such as resistin and leptin, is significantly more useful in predicting patients who will eventually be diagnosed with GDM in their 24th through 28th weeks. The same study also revealed the data for visfatin levels when predicting GDM in the 24th through 28th weeks with a sensitivity of 87.1% and a specificity of 70% (5). Our results for sensitivity and specificity are close, but they are less than what they observed. However, we think lower values can be associated with decreased passage from serum to saliva or the gestational week differences during the visfatin collection period.

In conclusion, in the search for a simple, cost-effective, non-invasive, patient-friendly technique, saliva must be considered as a specimen because it is easy to collect, simple to use, and requires no invasive collection procedures. To the best of our knowledge, this study is the first to use visfatin adipokine to diagnose GDM using saliva samples. In our study, we believe that observing a significant difference in visfatin values among patients with GDM was a big step. However, we were unable to establish a sensitivity and specificity rate high enough to replace OGTT for GDM testing. Even so, with further studies on the horizon that incorporate analyzing larger samples of the population and other molecules, especially other adipokines, physicians’ preferences might shift toward saliva testing instead of OGTT.
